# A Systematic Review of Statins for the Treatment of Nonalcoholic Steatohepatitis: Safety, Efficacy, and Mechanism of Action

**DOI:** 10.3390/molecules29081859

**Published:** 2024-04-19

**Authors:** Shiqin Zhang, Xiaoling Ren, Bingzheng Zhang, Tian Lan, Bing Liu

**Affiliations:** 1 School of Pharmacy, Guangdong Pharmaceutical University, Guangzhou 510006, China; zhangshiqin233@163.com (S.Z.); r17071124@163.com (X.R.); zbz012345@163.com (B.Z.); 2 Department of Pharmacology, College of Pharmacy, Harbin Medical University, Harbin 150086, China

**Keywords:** nonalcoholic steatohepatitis (NASH), statins, cardiovascular risk, drug safety, molecular mechanism

## Abstract

Nonalcoholic fatty liver disease (NAFLD) is the liver component of a cluster of conditions, while its subtype, nonalcoholic steatohepatitis (NASH), emerges as a potentially progressive liver disorder that harbors the risk of evolving into cirrhosis and culminating in hepatocellular carcinoma (HCC). NASH and cardiovascular disease (CVD) have common risk factors, but compared to liver-related causes, the most common cause of death in NASH patients is CVD. Within the pharmacological armamentarium, statins, celebrated for their lipid-modulating prowess, have now garnered attention for their expansive therapeutic potential in NASH. Evidence from a plethora of studies suggests that statins not only manifest anti-inflammatory and antifibrotic properties but also impart a multifaceted beneficial impact on hepatic health. In this review, we used “statin”, “NAFLD”, “NASH”, and “CVD” as the major keywords and conducted a literature search using the PubMed and Web of Science databases to determine the safety and efficacy of statins in patients and animals with NASH and NAFLD, and the mechanism of statin therapy for NASH. Simultaneously, we reviewed the important role of the intestinal microbiota in statin therapy for NASH, as it is hoped that statins will provide new insights into modulating the harmful inflammatory microbiota in the gut and reducing systemic inflammation in NASH patients.

## 1. Introduction

Nonalcoholic fatty liver disease (NAFLD) has emerged as the pre-eminent chronic hepatic disorder and is now the most rapidly escalating cause of liver-related mortality worldwide. NAFLD affects a quarter of the global adult populace, as the prevalence of NAFLD has soared from 25.3% in the span of 1990–2006 to an alarming 38.0% in the period of 2016–2019, and is expected to be the leading cause of liver transplantation by 2030. Within this cohort, about one-third of patients are grappling with nonalcoholic steatohepatitis (NASH). This condition will impose a substantial fiscal impact on healthcare systems [1,2,3]. Currently, in terms of the progress of research in the field of NASH, Resmetirom has been formally approved by the FDA for the treatment of adults with noncirrhotic NASH with moderate to advanced liver scarring (fibrosis). Although encouraging, the results raise important questions, such as the uncertainty about the overall risks and benefits of Resmetirom [4].

NAFLD is the hepatic manifestation of the metabolic syndrome, delineated by the accrual of hepatic lipids in the absence of excessive alcohol consumption [5]. It is a dynamic and progressive phenomenon that commences with the augmented accretion of fatty acids and triglycerides (>5%) in hepatocytes, leading to hepatic inflammation and culminating in hepatic injury [6]. NASH is a pathologic entity involving hepatocyte ballooning and lobular inflammation [7]. Once hepatic inflammation is triggered in NASH, it is perpetuated by several vicious cycles that occur both outside and inside the hepatic injury, which eventually result in cirrhosis and hepatocellular carcinoma (HCC) [8]. In addition to liver disease and mortality, NAFLD is associated with cardiovascular disease (CVD), metabolic syndrome, type 2 diabetes, and malignant tumors [2]. Therefore, not only is the deleterious impact of NAFLD related to the progression of hepatic deterioration but also heightens the independent risk of the development of atherosclerosis and other CVD-related morbidities [9]. Increased de novo lipogenesis (DNL) promotes liver fat accumulation in NAFLD, contributing to the development of a proatherosclerotic lipid profile and atherosclerotic cardiovascular disease [10]. Interestingly, certain novel NASH drugs, which promote weight reduction and ameliorate dyslipidemia and insulin resistance, may improve long-term clinical outcomes beyond their hepatic benefits by providing extended cardioprotective benefits [11].

There has been a notable rise in the administration of statins to individuals suffering from cardiovascular disease over the past few decades. Statins act by decreasing the cellular cholesterol content by selectively impeding the enzyme 3-hydroxy-3-methylglutaryl coenzyme A (HMG-CoA) reductase, curtailing cholesterol biosynthesis and diminishing hepatic cholesterol concentrations [12]. Excessive cholesterol accumulation in the liver or bloodstream can precipitate pathological conditions such as hepatic steatosis and atherosclerosis. Disrupted hepatic cholesterol equilibrium and the aggregation of free cholesterol are intricately associated with the pathogenesis of NASH/NAFLD [13,14,15]. Consequently, impaired hepatic cholesterol homeostasis may represent a common pathogenesis of both NAFLD and atherosclerosis [16]. The lipid-lowering capabilities of statins hold promise for conferring beneficial effects in NAFLD.

In recent years, the intestinal microbiota has emerged as a hot topic of research. The gut–liver axis refers to the bidirectional relationship between the gut and its resident microbiota, involving the transport of gut-derived metabolites via the portal vein to the liver, while bile and antibody secretion from the liver provide feedback to the intestine. A high-fat diet alters the microbiome, which subsequently compromises the integrity of both the intestinal barrier and the gut vascular barrier, and it facilitates the portal influx of bacterial products, exacerbating non-hepatic inflammation and metabolic abnormalities [17]. Moreover, the intestinal microbiota presents potential as a predictive tool for individual responses to statin therapy. Dietary interventions inclusive of probiotics may enhance the efficacy of statins in managing hyperlipidemia, while concurrently mitigating adverse effects such as myopathy [18]. It is worth noting that concurrent administration of antibiotics with lovastatin may attenuate the systemic bioavailability of lovastatin’s active metabolites, thereby diminishing its therapeutic efficacy [19].

In this review, we outline the role of statins in the management of NASH and assess their safety. The mechanism of statins in the treatment of NASH has been demonstrated through Kupffer cells (KCs), hepatic stellate cells (HSCs), liver sinusoidal endothelial cells (LSECs), Paraoxonase 1 (PON1), small guanine triphosphate binding proteins (GTPases), Peroxisome proliferator-activated receptors (PPARs), AMP-activated protein kinase (AMPK) and ferroptosis. We next summarize the most recent advancements in understanding the modulation of NASH and CVD pathogenesis by statins via the intestinal microbiota, which may provide new potential therapeutic targets for NASH.

## 2. Hypolipidemic and Pleiotropy of Statins

### 2.1. Hypolipidemic

Statins are used to treat lipid disorders caused by elevated cholesterol in the world and are a hallmark of liposuction therapy. As reductase inhibitors of HMG-CoA, statins curtail cholesterol formation by targeting and impeding a key step in the biosynthesis of isoprenoids and sterols, effectively lowering the serum cholesterol levels, which significantly reduces the morbidity and mortality of CVD [20]. One of the main mechanisms by which statins exert their therapeutic effect is promoting lipoprotein clearance, primarily by upregulating low-density lipoprotein receptor (LDL-R) expression without affecting very low-density lipoprotein (VLDL) synthesis [21], and reducing the content of low-density lipoprotein cholesterol (LDL-C) and the risk associated with CVD [22]. This multifaceted approach places statins at the forefront of interventions combating lipid-related CVD ailments. Research has shown a link between the impact of statins on reducing LDL-C levels and the duration of drug usage. In the case of short-acting statins, there was a notable increase in LDL-C and total cholesterol (TC) reduction during the evening dose when compared to the morning [23]. In addition to lowering LDL-C, statins exhibit a broader metabolic influence by reducing the fasting blood cholesterol and triglyceride (TG) concentrations. Notably, prolonged statin therapy moderates the postprandial TG elevation typically induced by lipid-rich meals [24]. Statins can raise the high-density lipoprotein cholesterol (HDL-C) levels, and cause low levels of HDL-C that are associated with an increased risk of coronary heart disease (CHD) events [25]. Such findings accentuate the relationship between statin administration and lipid profile optimization, supporting their indispensable status in contemporary cardiovascular therapeutics.

### 2.2. Pleiotropy

Statins are increasingly recognized for their multifaceted roles, which extend beyond their primary function of lowering LDL. This breadth of action, known as ‘statin pleiotropy’, has catalyzed a burgeoning research interest, deepening our understanding of statins’ comprehensive impact on CVD [26]. High-sensitivity C-reactive protein (Hs-CRP) is an inflammatory biomarker that predicts vascular risk with an effect estimate paralleling TC or HDL-C [27]. Statin treatment may reduce vascular inflammation as assessed by Hs-CRP, and higher Hs-CRP levels appeared to increase the risk of recurrent stroke and vascular events [28,29]. HMG-CoA reductase inhibition leads to an upsurge in endothelial nitric oxide (NO) synthase activity, thereby enhancing the bioavailability of NO; thus, statins may stabilize atherosclerotic plaques by increasing the bioavailability of NO [30,31]. Statins have also been demonstrated to enhance endothelial function and to attenuate both platelet reactivity and the production of proinflammatory cytokines, facilitating the recovery of endothelial function after myocardial ischemia–reperfusion injury in a dose-dependent manner [32,33]. These pleiotropic effects may be attributed to the inhibition of synthesis of isoprenoids, which are important lipid attachments for intracellular signaling molecules [34,35]. The accumulating body of research continues to confirm the diverse benefits of statin pleiotropy, progressively illuminating its efficacy far beyond the confines of CVD management.

## 3. Mechanisms Linking NAFLD and CVD

### 3.1. Insulin Resistance

**Figure 1:**
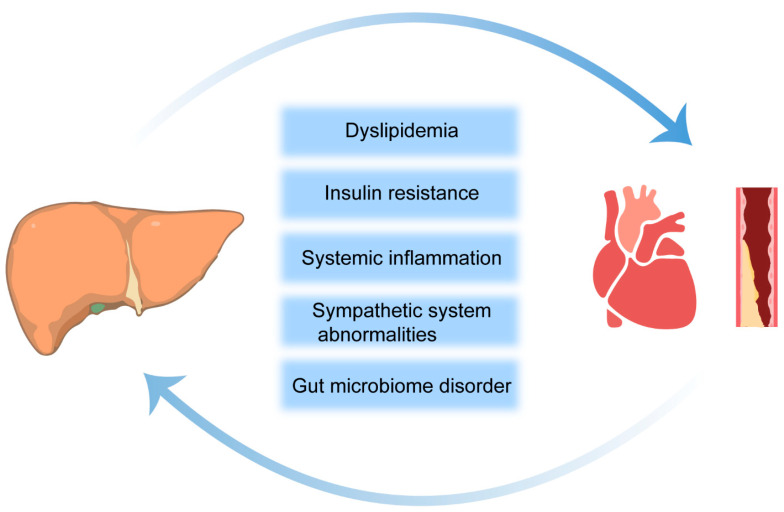
Potential risk factors linking NAFLD to the development and progression of CVD. The arrows indicate that NAFLD and CVD are interconnected.

Over the past 30 years, there have been many new insights into the pathogenesis of NAFLD. There is a partial relationship of the etiology between NAFLD and CVD, and the most common cause of death in patients with NAFLD is CVD rather than chronic liver disease [36]. The precise pathophysiological mechanisms that elevate the CVD risk in the context of NASH/NAFLD are yet to be fully elucidated. However, current evidence points to a constellation of potential factors, including dyslipidemia, insulin resistance (IR), systemic inflammation, coagulopathies, anomalies within the sympathetic nervous system, and perturbations in the gut microbiome. Among these, dyslipidemia, IR, and systemic inflammation stand out as conventional risk factors for CVD and as being independent of NASH/NAFLD in terms of exacerbating the CVD risk (Figure 1) [37].

IR is recognized as a pivotal factor in the evolution from simple fatty liver to NASH and hepatic cirrhosis, which also poses a significant risk of CVD. IR-induced compensatory hyperinsulinemia also engenders a spectrum of metabolic and cellular aberrations, including dyslipidemia, hypertension, endothelial dysfunction, oxidative stress, and alterations in cardiac metabolism [38]. Chronically elevated levels of serum glucose, especially postprandial glucose spikes, instigate sympathetic hyperactivity and the synthesis of advanced glycation end products (AGEs). Through their interaction with specific receptors, AGEs activate proinflammatory signaling cascades and foster oxidative stress, chronic vascular inflammation, endothelial dysfunction, lipoprotein metabolism disorders, and the accumulation of ectopic fat [39,40]. Hyperinsulinemia alters the transport of intracellular free fatty acids (FFAs) and transfers fatty acid uptake from adipose tissue to other metabolic organs, thereby contributing to the association of the ectopic fat distribution with obesity-related metabolic diseases. Furthermore, impaired insulin metabolism in these organs can lead to the exacerbation of IR, which in turn triggers a cascade of cardiometabolic dysfunction in the heart [41]. Consequently, the aggravation of IR in NASH/NAFLD patients creates favorable conditions for atherosclerotic dyslipidemia, inducing cardiomyopathy and arrhythmias, ultimately leading to CVD [42].

### 3.2. Dyslipidemia

Dyslipidemia encompasses a broad spectrum of lipid perturbations and manifests as increased plasma TG and LDL-C and reduced levels of HDL-C. It may increase the risk of CVD in patients with NASH/NAFLD [43]. Furthermore, the severity of NASH/NAFLD is positively correlated with the concentrations of small dense low-density lipoprotein (sdLDL) and the ratio of sdLDL/LDL. The study also found that the cholesterol content within sdLDL is linked to an elevated risk of CVD outcomes across various vascular territories [44,45]. In NAFLD, the mechanisms of hepatic lipid acquisition, predominantly through increased fatty acid uptake and DNL, are enhanced even in the presence of steatosis. While lipid disposal mechanisms may be upregulated, they are ultimately insufficient to offset the progressive accumulation of intrahepatic fat [46]. These changes lead to lipid dysregulation, which not only causes hepatocyte apoptosis and necrosis but also increases the possibility of CVD in patients with NASH/NAFLD.

### 3.3. Localized and Systemic Inflammation

NAFLD is understood as a systemic metabolic disorder, with its inflammatory triggers emanating not only from within the hepatic milieu through lipotoxicity, innate immune responses, and cell death pathways but also from external sources such as adipose tissue and the gut [8]. The systemic chronic inflammatory state is fueled by the reciprocal exchange of inflammatory mediators among the liver, adipose tissue, and the gut [47]. Fetuin-A, synthetized in the liver, is associated with endothelial dysfunction and an increased risk of ischemic stroke and carotid atherosclerosis, particularly when it provokes mild inflammation. Furthermore, elevated serum levels of C-reactive protein (CRP) have been correlated with carotid atherosclerosis, attributable to CRP’s role in enhancing plasminogen expression, facilitating the adhesion of molecules to endothelial cells, and augmenting macrophage LDL phagocytosis [48]. Hepatocyte-derived extracellular vesicles (EVs) from fatty livers notably instigate endothelial inflammation. MicroRNA-1 (miR-1), a critical component within these EVs, which triggers endothelial cell inflammation by downregulating Krüppel-like factor 4 (KLF4) and activating the nuclear factor kappa-B (NF-κB) pathway. Interestingly, inhibiting miR-1 not only reduces endothelial inflammation in vitro but also mitigates atherogenesis in ApoE-deficient mice [9]. Due to being central to the nexus between NASH and CVD, the hepatic miRNAs can enter into the bloodstream and promote CVD through alterations in lipid metabolism and/or the promotion of systemic inflammation [49].

## 4. Statin Therapy in Patients with NAFLD: Safety and Efficacy

### 4.1. Effects of Statins on NAFLD Patients with CVD

NAFLD and CVD have common risk factors. In NAFLD, the increased liver-related mortality is primarily attributed to complications of advanced liver fibrosis and cirrhosis, such as HCC and decompensated cirrhosis. Compared to liver-related causes, however, most patients with NAFLD face a higher risk of early morbidity and mortality from CVD [50,51]. In addition, the risk of CVD and liver-related mortality rises exponentially rather than linearly as liver fibrosis progresses [52]. Statins, mimicking HMG-CoA, the natural substrate, competitively inhibit the HMG-CoA reductase enzyme, thereby reducing mevalonate production and downstream cholesterol biosynthesis. Considering that most cholesterol synthesis occurs in hepatocytes, HMG-CoA reductase inhibitors primarily target the liver [53]. Dyslipidemia and NAFLD usually coexist, and pooled estimates indicate a 69.16% prevalence of hyperlipidemia/dyslipidemia among NAFLD and NASH patients [36]; therefore, statins can often be used as primary and secondary prophylaxis for CVD in patients with NAFLD. The current statin treatments in NASH/NAFLD clinical trials are summarized in Table 1.

Three post hoc analyses of randomized controlled trials (*n* = 1600, *n* = 1123, *n* = 8864) indicate that atorvastatin is beneficial for NASH/NAFLD on the basis of a reduction in liver enzymes and improvements in ultrasonography. Moreover, statin treatment halved the CVD morbidity and mortality, as well as reduced the CVD events by two-thirds, compared to NASH/NAFLD patients not receiving statins [55]. Another large-scale cross-sectional analysis within a population-based cohort study found that statin-naive individuals with suspected NAFLD (Fatty Liver Index ≥ 60) and suspected advanced fibrosis (NAFLD fibrosis score > 0.676) face a significantly elevated cardiovascular risk, thereby warranting a higher consideration for statin therapy. The proportion of NASH subjects requiring statin treatment increases with increasing LDL-C levels and higher CVD risk prediction categories [30].

In summary, statin therapy has a significant ameliorative effect in patients with NASH/NAFLD. Most importantly, statins are beneficial for their concurrent CVD. As the disease worsens, statins may have the potential to save the lives of patients with severe liver disease complicated by CVD.

### 4.2. Safety Assessment of Statins in the Treatment of NASH/NAFLD

Many lipid-lowering drugs have side effects, including elevated liver function and hepatotoxicity. Statins, as lipid-lowering drugs, have also gained significant attention for their potential toxicity. A systematic examination and meta-analysis of over 90,000 participants in randomized studies revealed that statins markedly elevate both the relative and absolute risks of myopathy, kidney, and liver dysfunction [56]. Myotoxic effects caused by statins rank among the most frequent side effects, presenting as exhaustion, muscle pain, muscle weakness, nocturnal cramping, and more, with occurrence rates varying between 7% and 29% [57]. Furthermore, randomized studies indicate that moderate-intensity statins elevate the risk of type 2 diabetes by approximately 11%, and there is a potential additional 12% risk increase when moving to high-intensity statins [58]. Research indicates that statin prescription is sometimes limited in NASH/NAFLD patients due to the concern of physicians about hepatotoxicity [59]. In fact, statins have only a slight effect on aminotransferases, and they even have a decreasing effect on these enzymes. The Greek Atorvastatin and Coronary Heart Disease Evaluation (GREACE) study revealed that of 437 patients presenting with moderately abnormal liver tests at baseline, potentially indicative of NAFLD, 227 treated with a statin (predominantly atorvastatin) exhibited significant improvements in the liver function tests. In contrast, the 210 patients not receiving statin treatment experienced further elevations in the liver enzyme concentrations [60].

Different clinical studies have consistently demonstrated that the serum aminotransferase levels are reduced significantly in NAFLD patients with dyslipidemia by atorvastatin and simvastatin treatment [61,62]. Although prolonged use of certain drugs, such as rosuvastatin, can cause liver enzyme abnormalities, none of the subjects exhibited more than a threefold increase in these enzymes, and they showed significant improvements in lipid parameters [63]. A recent meta-analysis scrutinized variations in the liver function tests, specifically alanine transaminase (ALT), aspartate transaminase (AST), and gamma-glutamyl transpeptidase (γ-GGT), in 22 studies with 2345 NAFLD patients treated with statins. The results reveal a significant diminution in the liver enzyme levels in the statin-treated NAFLD patients [64]. Another meta-analysis similarly indicated that statins reduced the liver enzyme levels and improved liver histology [65]. The prospective study investigating statin therapy’s impact on liver enzyme in the very elderly (≥80 years old) included 515 patients aged 80 to 98 with hypercholesterolemia (LDL-C levels ≥3.4 and <5.7 mmol/L), atherosclerosis, CHD, or a CHD-risk equivalent. The findings indicate that 24 patients (4.7, 95% CI 2.7–6.6) experienced increased hepatic aminotransferase levels [66], implying that treatment with statins is safe, even in elderly patients. Statins are deemed safe for patients with cirrhosis. A retrospective cohort study of veterans demonstrated that statin therapy reduced the risk of acute-on-chronic liver failure in cirrhotic patients [67]. Furthermore, statin therapy significantly reduces the risk of HCC in patients with diabetes, NASH, and cirrhosis [68,69].

In 2006, the Liver Expert Panel of the National Lipid Association (NLA), in a series of special reports on the safety of statins, indicated that elevated liver enzymes do not necessarily lead to hepatotoxicity. Additionally, patients with suspected NAFLD and elevated liver enzymes do not exhibit an increased risk of developing liver injury when using statins compared to those with normal liver enzymes prior to recruitment [70]. The 2020 Evidence-Based Clinical Practice Guidelines for NAFLD/NASH also advocate for the consideration of statins in NAFLD/NASH patients with hypercholesterolemia [71]. A large amount of evidence in recent years suggests that statin therapy for NASH has a low risk of causing serious hepatotoxic reactions, prompting an increase in the use of statins for primary prevention over time [59,72].

## 5. Effects of Statins on Liver Histology—From Animal Models to Human Studies

### 5.1. Improvement of Steatosis

NAFLD is typified by hepatic steatosis, marked by over 5% parenchymal fat accumulation, without hepatocyte injury [7]. Statins, as efficacious lipid-lowering agents, reduce LDL-C by 20–60%, TG by 10–33%, and increase HDL-C by 5–10% in NAFLD patients [73]. Statins may potentially contribute to blocking NAFLD progression by controlling lipid status. Specifically, atorvastatin has been shown to effectively ameliorate NAFLD-related hyperlipidemia in rats, reducing liver steatosis and modulating the expression of lipid metabolism-regulating genes [74]. Likewise, the treatment of NAFLD rats with simvastatin and fluvastatin reduced their liver weight, hepatic index, ALT, AST, and regulated abnormal lipid metabolism [75,76].

**Figure 2:**
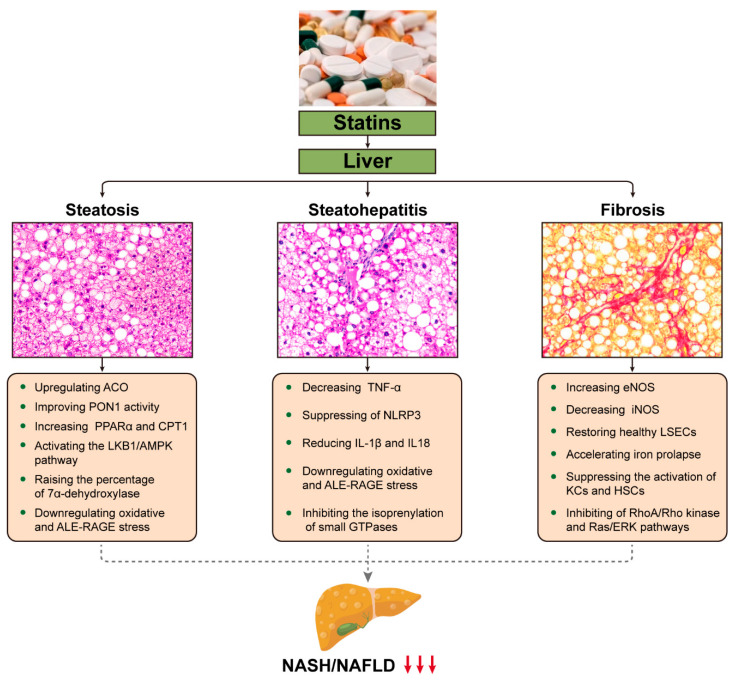
Potential mechanisms by which statins may favorably affect liver histology in NASH/NAFLD. This cartoon emphasizes the pleiotropic effects of statins on the liver and the potential mechanisms that may combine to ameliorate the primary pathological conditions of NASH/NAFLD, such as steatosis, inflammation, and fibrosis. The downward pointing arrow signifies downregulation. ACO, acyl-CoA oxidase; PON1, paraoxonase 1; PPAR, peroxisome proliferator-activated receptor; CPT, carnitine palmitoyltransferase; LKB1/AMPK, AMP-activated protein kinase; ALE-RAGE, advanced lipoxidation end product-receptors of advanced glycation end products; TNF, tumor necrosis factor; NLRP3, NOD-like receptor family pyrin domain-containing 3; IL-1β, cytokines interleukin (IL)-1β; GTPases, small guanine triphosphate-binding proteins; eNOS, endothelial nitric oxide synthase; iNOS, inducible nitric oxide synthase; LSECs, liver sinusoidal endothelial cells; KCs, Kupffer cells; HSCs, hepatic stellate cells; RAS/ERK; Ras-extracellular signal-regulated kinase.

In an open-label trial, 70 participants with NAFLD were randomized to receive either ezetimibe combined with rosuvastatin or rosuvastatin alone for a duration of up to 24 weeks, and the effectiveness of ezetimibe plus rosuvastatin was assessed compared to rosuvastatin alone in reducing hepatic fat by utilizing magnetic resonance imaging-derived proton density fat fraction (MRI-PDFF) in patients. The results demonstrated that hepatic steatosis was reduced by the combination or individual use of rosuvastatin [77]. Another study found that during 12 weeks of continuous treatment with either pitavastatin (2–4 mg/day) or atorvastatin (10–20 mg/day), CT scans showed no significant elevation in ALT and a significant reduction in hepatic fat accumulation (pitavastatin group: *p* = 0.014; atorvastatin group: *p* = 0.021) during treatment [78]. In 2015, Kargiotis et al. examined patients treated with rosuvastatin (10 mg/d) monotherapy for 12 months, with repeat liver biopsy and ultrasound at the end of treatment. It was found that the NASH was improved in 95% of the patients [79]. Several studies have indicated that statins can reduce hepatic steatosis in patients [80,81,82]; however, partial statin use, such as pitavastatin, has no significant effect on liver steatosis [83]. Long-term treatment with statins (18 months) did not translate into a histologic improvement in the NAFLD Activity Score (NAS), despite a reduction in liver fat (13 ± 2 vs. 8 ± 2%, *p* < 0.001) [84]. The results and potential hepatoprotective mechanisms of statins used in NASH/NAFLD are listed below (Figure 2 and Table 2).

### 5.2. Reduction of Inflammation

Statins have some anti-inflammatory activity, with evidence showing their ability to attenuate the inflammatory and pathogenic activities of T cells via KLF2-dependent mechanisms [87]. Simvastatin prevented microcirculatory dysfunction and NAFLD by downregulating oxidative stress and advanced lipoxidation end product-receptors of advanced glycation end product (ALE-RAGE) stress, thus ameliorating steatosis, fibrosis, and inflammatory parameters [86]. Atorvastatin notably inhibits the activation of the NOD-like receptor family pyrin domain-containing 3 (NLRP3) inflammasome pathway triggered by cholesterol crystals, subsequently reducing the expression of pro-inflammatory cytokines IL-1β and IL-18, which are implicated in NAFLD progression. Atorvastatin has been found to inhibit the activation of the NOD-like receptor family, NOD-like receptor family pyrin domain-containing 3 (NLRP3) inflammasome pathway induced by cholesterol crystals, thereby reducing the expression of pro-inflammatory cytokines interleukin (IL)-1β and IL-18, which are implicated in NAFLD progression [85]. Additionally, atorvastatin also impedes NASH progression, partly by reducing tumor necrosis factor-α (TNF-α). The study enrolled 42 biopsy-proven nonalcoholic steatohepatitis patients with dyslipidemia. A 12-month treatment with atorvastatin (10 mg/day) resulted in significant decreases in the levels of AST, γ-GGT, LDL-C, TG, type IV collagen, and TNF-α, and it revealed that the extent of the LDL-C reduction by atorvastatin was independently associated with the increase in the liver to spleen density ratio [88]. Another study similarly found that NAFLD patients taking statins had a significant reduction in the necro-inflammatory phase of the liver [72]. Long-term statin therapy has been shown to improve steatosis, hepatocellular ballooning degeneration, and diffuse lobular mixed acute and chronic inflammation in 95% of patients based on repeat liver biopsy [79].

### 5.3. Improvement of Fibrosis

Advanced liver fibrosis serves as a pivotal prognostic determinant for end-stage liver disease, cardiovascular, and overall mortality [89]. Some studies have found that statins can be an effective antifibrotic agent in hepatic fibrosis [90,91,92]. For instance, simvastatin has been observed to enhance the prognosis of NASH-related fibrosis by modulating the expression of endothelial nitric oxide synthase (eNOS) and inducible nitric oxide synthase (iNOS) while inhibiting the activation of HSCs [93]. Fluvastatin alleviates steatosis-induced activation of HSCs and hepatic fibrogenesis by mitigating inflammation and oxidative stress in vitro and in vivo [75]. In addition, a clinical investigation, utilizing liver stiffness measurements (LSM) as an index of fibrosis, discerned that statin administration correlates with a reduced likelihood of advanced liver fibrosis (OR 0.35, 95% CI 0.13–0.90, *p* = 0.03) [94].

In a cross-sectional analysis involving 346 diabetics with biopsy-confirmed NAFLD, statin use was independently and inversely correlated with both NASH (OR 0.57, 95% CI 0.32 to 1.01, *p* = 0.055) and significant fibrosis (SF) (OR 0.47, 95% CI 0.26 to 0.84, *p* = 0.011) [95]. Sfikas et al. studied 604 military personnel with NASH/NAFLD to assess the effects of diet–exercise, and one year of treatment with atorvastatin, rosuvastatin, or pitavastatin, on NASH/NAFLD. The results showed that all three statins reduced NASH and the Fibrosis-4 score (FIB-4) [96]. Previous studies have indicated that statins may not improve liver fibrosis [81,97,98]; however, a recent study found that statin use reduced the risk of significant liver fibrosis (AOR 0.43; 95% CI 0.42–0.44) in a national case-control study [99], and the risk of HCC was lower in patients with NASH and advanced fibrosis after statin use [100,101].

## 6. Mechanisms of Statins in the Treatment of NASH/NAFLD

### 6.1. KCs, HSCs and LSECs

In atherosclerosis and NASH, the inflammatory response is primarily driven by lysosomal cholesterol accumulation in macrophages rather than the overall amount of intracellular lipids [102]. In animal models, dietary cholesterol can activate hepatic injury by promoting the M1 phenotype in KCs, leading to the activation of HSCs, oxidative stress, and inflammation [103]. The KCs can be activated by free cholesterol (FC), cholesteryl esters and TG around dead hepatocytes to form coronary structures, and a liver with these activated KCs progresses into inflammation and fibrosis distinct from simple steatosis [104]. Paracrine signaling from various resident and inflammatory cells, including hepatocytes, LSECs, hepatic macrophages, natural killer/natural killer T cells, biliary epithelial cells, hepatic progenitor cells, and platelets, exerts a direct or indirect regulatory influence on the differentiation and activation of HSCs. Activated HSCs are pivotal in the initiation and progression of liver fibrosis [105]. Furthermore, altered LSECs contribute to hepatic angiogenesis, inflammation, fibrosis and HCC during the stages of NASH. Multiple pieces of evidence indicate that the dysfunction of LSECs in fatty livers is associated with increased intrahepatic vascular resistance related to steatosis [106].

Statins reduce hepatocellular cholesterol uptake, effectively preventing or reversing cholesterol accumulation in hepatocytes, and they inhibit the activation of KCs and HSCs, which slows down liver fibrosis [107]. A study showed that atorvastatin reduced hepatic steatosis and inflammation induced by a high-saturated fat, high-fructose, and high-cholesterol diet in animals with NASH and significantly regulated bile acid metabolism by altering intestinal reabsorption and hepatic synthesis in NASH mice [108]. The separation of the components in an atherogenic diet has revealed that liver damage and fibrosis may be attributed to the initial accumulation of bile acid. Bile acids play a role in the transdifferentiation of HSCs into myofibroblasts, ultimately resulting in fibrosis, which suggests that hepatic cholesterol and bile acid homeostasis may influence fibrosis caused by HSCs proliferation [109].

Moreover, combination therapy with lipid-lowering drugs has proven more efficacious in treating NASH compared to individual drug use. In multiple animal model studies, investigators have evaluated the roles of different interventions in NASH, confirming that an ezetimibe/atorvastatin combination normalized the hepatic FC levels, although it had a minimal impact on saturated FFAs and other lipids. Lipid-lowering drugs can break down cholesterol crystals, crown-like structures of activated KCs and improve fibrosis. In contrast, ezetimibe and atorvastatin alone yield similar but less pronounced effects [110,111]. Increased activation of KCs and HSCs due to the dysfunction of LSECs may be modulated by statins. A study of rats treated with simvastatin or atorvastatin demonstrated that statins not only improve NASH histology but also restore healthy LSECs and HSCs phenotype, leading to decreased portal pressure and, consequently, an improved prognosis for the disease [112].

In summary, statins counteract hepatic lipotoxicity by processing cholesterol crystals and KCs with a coronal structure, further preventing inflammation and fibrosis formation, restoring healthy LSECs, and inhibiting HSCs activation, thus antagonizing the development of fibrosis in NASH.

### 6.2. PON1

PON1 is an antioxidant enzyme of hepatic origin. The structure activity of three groups of known substrates of PON1 (phosphotriesters, esters, and lactones) has been verified and PON1 is, in fact, a lactonase [113]. PON1 hinders oxidative modification of LDL through the elimination of lipid peroxides from LDL. PON1 is recognized as an essential component of the antioxidant and anti-inflammatory functions of HDL. The serum activity of PON1 correlates with systemic lipid peroxidation (LPO) stress and potential cardiovascular risk [114,115]. A meta-analysis found that paraoxonase activity, which is related to PON1, was markedly diminished in patients with NAFLD compared to those without the condition. This suggests that the paraoxonase levels may impact the pathophysiology of NASH/NAFLD and could serve as a useful biomarker for the condition [116].

Statins have been demonstrated to restore the serum PON1 levels in NASH/NAFLD patients. Increased PON1 activity attenuates NASH/NAFLD-induced LPO. In a clinical trial, 50 NAFLD patients were bifurcated into 2 cohorts, 1 with an 8-month intervention of 40 mg tablet atorvastatin and 1 without atorvastatin. Various investigations were conducted, including abdominal ultrasonography, serum PON1 activity level, liver function tests, serum lipid profile, and serum levels of malondialdehyde (MDA), an indicator of oxidative stress. The results showed that after atorvastatin treatment, the serum PON1 activity was significantly increased, the blood lipids, especially LDL-C, were decreased, hepatic oxidative stress was reduced and the progression of NAFLD was reversed [117].

However, whether statins can ameliorate NASH by increasing serum PON1 activity has not been reported in animals, and there are only a few studies even in clinical trials. If it is found in animal models that PON1 is the therapeutic target of statin action in NASH, it will be better established.

### 6.3. GTPases

The Rab family GTPases are quintessential controllers of vesicle transport and membrane trafficking in eukaryotic cells. They interact with various effectors, such as molecular motors, scaffolding proteins, and lipid kinases, orchestrating virtually every aspect of vesicular trafficking across cellular compartments [118]. The regulation of intracellular trafficking and the interactions with regulators and effectors of Rho proteins rely heavily on the post-translational isoprenylation of small G proteins. Statins impede the production of isoprenoid intermediates by restraining mevalonate synthesis, thus hindering the isoprenylation of small GTPases and consequently inhibiting their signaling. Statins also attenuate hepatic inflammation and fibrosis by inhibiting signaling downstream of the Rho family small G proteins (RhoA, RAS). The cause of this effect can be attributed to the multiple actions of statins rather than their ability to lower cholesterol [119,120,121,122].

Researchers fed 12-week-old ApoE^−/−^ mice a Western-style diet for 7 weeks to induce NASH, while treating the mice with simvastatin for 6 weeks. The results revealed that the antifibrotic and anti-inflammatory effects of simvastatin were dependent on the inhibition of two specific pathways: the Ras/ERK1/2 (Ras-extracellular signal-regulated kinase) pathway and the RhoA/Rho kinase pathway. These findings highlight the potential therapeutic benefits of statins in the treatment of NASH and provide insights into the underlying mechanisms of its action [123].

### 6.4. PPARs

Peroxisome proliferator-activated receptors (PPARs), which bind fatty acids and their metabolites and control inflammatory and metabolic pathways, are also essential regulators of lipid and glucose metabolism, inflammation and fibrogenesis in various tissues [124]. PPARα is a key factor in regulating fatty acid metabolism and ketogenesis. It has the ability to regulate the transportation of fatty acids, oxidation of peroxisomes or mitochondria, and lipolysis, ultimately impacting the production of apolipoproteins [125]. Deletion of hepatocyte PPARα in NAFLD mice disrupted liver and systemic fatty acid homeostasis, leading to hepatic lipid accumulation [126]. Mice treated with different PPAR agonists improved both their steatosis and hepatitis [127]. Previously, statins have been found to exert anti-inflammatory effects via PPARα. The study found that the liver PPARα, acyl-CoA oxidase (ACO), and carnitine palmitoyltransferase I (CPTI) mRNA levels and hepatic fatty acid β-oxidation activity were increased by atorvastatin at 30 mg/kg [128]. Rosuvastatin treatment attenuated hepatic steatosis in NAFLD mice by modulating PPAR homeostasis. This effect was accompanied by a reduction in insulin resistance, an improvement in the anti-inflammatory adipokine profile, and a decrease in HSC activation [129]. Different types of statins (fluvastatin, pravastatin, simvastatin, atorvastatin, and rosuvastatin) can prevent NASH by inducing PPARα and increasing mitochondrial and peroxisomal fatty acid oxidation (FAO) [130].

### 6.5. AMPK

AMPK plays an essential role in controlling a variety of metabolic pathways. Its significance lies in its ability to regulate glucose uptake, uphold mitochondrial equilibrium, avert lysosomal harm, and facilitate autophagy. Furthermore, AMPK hinders the production of fatty acids and cholesterol [131,132]. AMPK, adept at inhibiting various enzymes and transcription factors pivotal for lipid biosynthesis, plays a critical role in metabolic regulation. Diminished AMPK activity impairs FAO and augments adipogenesis by attenuating ACC phosphorylation [133]. Fluvastatin was found to act as an SIRT6 agonist and inhibit lipid synthesis in hepatocytes by activating the LKB1/AMPK pathway to regulate the transcriptional factor Srebp1 [134]. In another study, atorvastatin also mitigated hepatic fat accumulation through AMPK-dependent upregulation of PPARα and PGC1α in the high-fat diet-induced NAFLD hamster model [135]. Additionally, simvastatin treatment led to the suppression of oncoproteins STAT3 and Skp2 and activation of the energy sensor AMPK, culminating in reduced tumor growth in HepG2 xenograft mice [136].

### 6.6. Ferroptosis

Ferroptosis represents an iron-dependent, non-apoptotic mode of cell death, characterized by large levels of LPO [137]. The liver plays a pivotal role in regulating the systemic iron balance through the synthesis and secretion of hepcidin, a major regulator of iron homeostasis [138]. Ferroptosis has been proposed to be implicated in the development and progression of NASH/NAFLD [139]. Knockout of the iron chaperone protein PCBP1 in hepatocytes resulted in defects in liver iron homeostasis and led to reactive oxygen species (ROS) production, which caused LPO and hepatic steatosis in mice [140]. Statins can induce ferroptosis and suppress tumor growth [141]. Subsequent research has elucidated that simvastatin could suppress the expression of HMG-CoA, consequently downregulating the mevalonate pathway and glutathione peroxidase 4 (GPX4), which in turn triggers ferroptosis in cancer cells [142]. Simvastatin also mediates the activation of HSCs through the mevalonate pathway, reducing the intracellular cholesterol levels in HSCs and inducing iron prolapse, and may be an potential therapeutic agent for treating hepatic fibrosis [143]. Although the effects of statins on the iron death pathways have been reported [144], whether one of the pathological mechanisms by which statins are favorable to the progression of NASH/NAFLD is linked to the inhibition of iron death requires further investigation.

### 6.7. Intestinal Microbiota

In 2011, Arumugam M and colleagues first proposed the concept of ‘enterotypes’ [145]. Later, scientists further proposed that the microbial composition of the human gut can be divided into four distinct enterotypes, such as *Bacteroidetes1* (Bact1), *Bacteroidetes2* (Bact2), *Prevotella* (Prev) and *Ruminococcaceae* (Rum) [146,147,148]. Sara et al. [148] indicated that less than 6% of obese individuals treated with statins had a Bact2 gut phenotype, which is comparable to non-obese individuals. It also suggests that statins may play a role in modulating the harmful inflammatory microbiota of the gut and alleviating the systemic inflammation levels in obese patients. These findings indicate that commonly used cholesterol-lowering statin drugs may be a potential therapeutic option for microbiota modulation (Table 3).

Cholesterol is oxidized in hepatocytes via cytochrome P450 to synthesize bile acids. The intestinal microbiota can biotransform these BAs into their unconjugated forms via bile salt hydrolase (BSH) activity, subsequently producing secondary BAs through reactions such as 7α-dehydroxylation or isopropylation [157]. Several bacterial genera, including *Bacteroides*, *Bifidobacterium*, *Clostridium*, *Lactobacillus,* and *Listeria,* have been identified as containing BSHs [158]. Increasingly, studies are focusing on the regulatory effects of statins on cardiovascular and other diseases by the intestinal microbiota. Such research found that the treatment of hypercholesterolemic mice with atorvastatin resulted in elevated levels of the anti-inflammatory bacteria [149]. Similarly, statins can significantly modify inflammation-associated bacterial flora, such as *Butyricimonas* and *Mucispirillum* [153]. Diminished populations of intestinal *Lactobacillus* and *Bifidobacterium*, leading to transport defects, may significantly impair the efficacy of rosuvastatin in reducing the serum levels of TC and LDL-C in rats [159]. Studies have also found that the hypolipidemic effect of simvastatin was related to the composition of the intestinal microbiota. Simvastatin stimulates hepatic Farnesoid X receptor (FXR) and increases Cyp7a1 gene expression in mice on an HFD diet, which was impaired by antibiotic treatment [160]. This further supports the therapeutic effect of statins on disease via the intestinal microbiota. The same was found in studies of ischemic stroke, showing that atorvastatin not only increased abundance of *Firmicutes* and *Lactobacillus* and increased the fecal butyrate level but also regulated intestinal immune function, significantly reduced the level of circulating endotoxin, and promoted intestinal barrier function [151]. Cross-sectional studies have also shown that long-term, regular oral administration of aspirin in conjunction with atorvastatin modulates the human gut microbiota, contributing to the prevention of ischemic stroke [161].

Development of fibrosis, cirrhosis and HCC in NASH/NAFLD patients is strongly associated with hepatic cholesterol metabolism [107,162]. Statins can prevent further progression of NAFLD by modulating the disorders of the intestinal microbiota and its metabolites, improving bile acid metabolism. A study proposed that an interaction of the intestinal microbiota and cholesterol metabolism leads to increased levels of adiposity in mice [163], and the presence of cholesterol-metabolizing bacteria correlates with reduced levels of TC in both the stool and serum in human cohorts [164]. There have been researchers who have associated statins with NAFLD through the intestinal microbiota [155]. They have found that high-fat/high-cholesterol (HFHC) led to intestinal microbiota in mice, such as *Mucispirillum*, *Desulfovibrio*, *Anaerotruncus* and *Desulfovibrionaceae* increased sequentially, while *Bifidobacterium* and *Bacteroides* were depleted, and impaired microbial tryptophan metabolism. Atorvastatin treatment reversed the abundance of disturbed intestinal microbiota, with increased serum taurocholic acid (TCA) and depleted 3-indolepropionic acid (IPA). The results revealed that atorvastatin completely prevented HFHC diet-induced NAFLD-HCC development [155]. In another study, atorvastatin was also found to be effective in reducing hepatic fat deposition in NASH mice by enhancing the percentage of 7α-dehydroxylase-expressing bacteria (*Ruminococcaceae*) in the intestine, which promotes the formation of deoxycholic acid and lithocholic acid, both of which are GPBAR1 agonists [156].

Despite this, there is still a deficiency of articles investigating the link between statins and NASH via the intestinal microbiota, and how statins ameliorate NASH through the intestinal microbiota and its metabolites still needs to be further explored.

## 7. Conclusions

**Figure 3:**
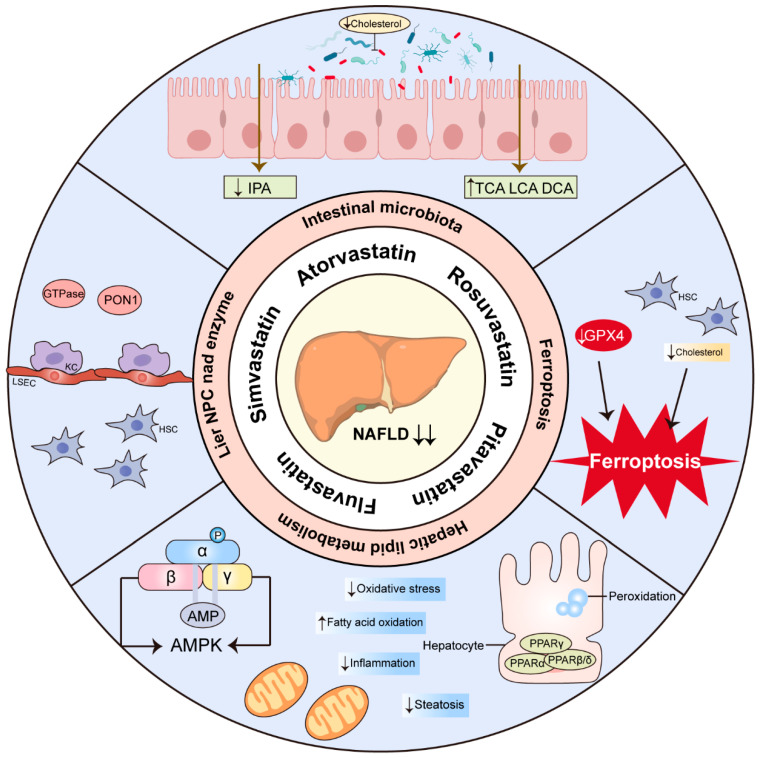
Overview of the proposed mechanisms of statin therapy for NASH. The arrows pointing up indicate upregulation and the pointing down indicate downregulation. IPA, indolepropionic acid; TCA, taurocholic acid; LCA, lithocholic acid; DCA, deoxycholic acid; GPX4, glutathione peroxidase 4; PPAR, peroxisome proliferator-activated receptor; AMPK, AMP-activated protein kinase; HSC, hepatic stellate cell; KC, Kupffer cells; LSEC, liver sinusoidal endothelial cells; PON1, paraoxonase 1; GTPase, small guanine triphosphate binding proteins; NPC, non-parenchymal cell.

NASH/NAFLD form part of a multisystemic disease that is considered to be the hepatic manifestation of metabolic syndrome and is a significant contributor to hepatic cirrhosis and HCC. Resmetirom received FDA approval on 14 March 2024. This marks it as the first medication approved for the treatment of NASH with liver fibrosis, providing a treatment option for these patients, in addition to diet and exercise. It also serves as a crucial reference for creating additional medications to treat NASH. As a lipid-lowering drug, statins have consistently demonstrated in several clinical trials having no significant effect on hepatotoxicity in patients, further encouraging the use of statins in NASH patients. Here, we propose that statins may be a core coordinator in orchestrating the lipids, inflammation, and fibrosis implicated in conditions associated with NASH. Multiple in vivo and in vitro research studies suggest that statins might prevent or treat NASH via HSCs, LSECs, PON1, GTPase, PPARs, AMPK, and ferroptosis. An increasing number of studies show that statins can improve CVD through the intestinal microbiota and have a protective effect on NASH (Figure 3). In this context, we need more clinical trials to explore the impact of statins on NASH patients and the cardiovascular risk mediated by the intestinal microbiota. The mechanism of statins in the treatment of NASH needs to be further delineated in view of the fact that more and more statin therapies have recently been identified.

## Figures and Tables

**Table 1:** Clinical trials for statins in treating NASH/NAFLD registered at ClinicalTrials.gov.

Study Title	Agent	Dose/Day (Statins)	Status	Major Inclusion Criteria	Estimated Completion	Trial Number
NAFLD pharmacological treatment: metformin versus atorvastatin	Metformin and atorvastatin	20 mg	Unknown, N/A	Signs of simple liver steatosis at ultrasonography	1 June 2015	NCT01544751
NAFLD influence of statin therapy	Rosuvastatin	/	Withdrawn, N/A	Cardiology patient fatty liver in a cohort of patients	1 September 2009	NCT00375349
Combination of obeticholic acid (OCA) and statins for monitoring of lipids (control) [54]	Obeticholic acid and atorvastatin	10 mg	Completed, phase 2	Histologic evidence of NASH	12 March 2018	NCT02633956
Atorvastatin versus vitamin E in treatment of NAFLD	Atorvastatin	20 mg	Unknown, N/A	Sign informed consent before involvement in any trial-related activity	1 December 2016	NCT01720719
Phase IV study to evaluate the effects of statin monotherapy or statin/ezetimibe combination therapy on hepatic steatosis in patients with hyperlipidemia and NAFLD	Rosuvastatin	5 mg	Completed, phase 4	Patients diagnosed with fatty liver or liver fibroscan	11 September 2019	NCT03434613
Atorvastatin, L-Carnitine and NASH	Atorvastatin, carnitine and atoral	20 mg	Unknown, N/A	NASH diagnosed on the basis of established criteria	1 December 2019	NCT01617772
Assessment of endothelial function in patients with NAFLD and the impact of statin treatment	Atorvastatin	20 mg	Withdrawn, N/A	Patients with fatty liver	1 December 2015	NCT01987310
Statins for the treatment of NASH	Atorvastatin	40 mg	Recruiting, phase 2	NASH or fibrosis stage ≥ 2	1 December 2024	NCT04679376
Effects of pitavastatin on insulin sensitivity and liver fat	Pitavastatin	4 mg	Completed, Unknown	BMI ≥ 27 kg/m^2^ and waist circumference ≥ 10^2^ cm, high probability risk factors for NAFLD	30 April 2018	NCT02290106
Liver cirrhosis network rosuvastatin efficacy and safety for cirrhosis in the United States	Rosuvastatin	20 mg	Recruiting, phase 2	Cirrhosis due to NASH, alcohol-associated liver disease, or chronic viral hepatitis or cryptogenic cirrhosis	1 November 2026	NCT05832229
Clinical effects of new approach on patients with NASH	Rosuvastatin, vitamin E, and N-acetyl cysteine	20 mg	Not yet recruiting, early phase 1	NASH diagnosis using fibroscan detecting the degree of steatosis and fibrosis	17 January 2025	NCT06105060
Comparative clinical study to evaluate the efficacy and safety of rosuvastatin vs CoQ10 on NASH	Coenzyme Q10 and rosuvastatin	20 mg	Not yet recruiting, phase 3	Patients have established diagnosis of NASH	1 April 2024	NCT05731596

A search of the keyword “NAFLD” or “NASH” in the item “Condition or disease” at https://clinicaltrials.gov/ (accessed on 5 November 2023) yielded 1404 listed studies, 12 of which are listed in this table that are related to statins. N/A, not available. NAFLD, nonalcoholic fatty liver disease; NASH, nonalcoholic steatohepatitis.

**Table 2:** Summary of studies evaluating the effects of statin use in NASH/NAFLD.

Type of Statin	Study Design	Diseases	Dose/Day	Results	Comments	Reference
Atorvastatin	Rat	NAFLD	30 mg/kg	Atorvastatin up-regulated the expression of PPARα, liver fatty acid β-oxidation, and reduced the liver TG	Atorvastatin treatment effectively improved NAFLD-related hyperlipidemia and inhibited liver steatosis, accompanied by modulating the expression of genes for regulating lipid metabolism	[74]
	Mice	NAFLD-NASH	4.5 mg/kg	Atorvastatin prevents cholesterol crystal formation, thereby precluding NLRP3 inflammasome activation to prevent further development of NAFLD	Atorvastatin prevents development of hepatic steatosis, inflammation and fibrosis in mice	[85]
	Human	Hypercholesteremia	10 mg	Atorvastatin reduced LDL-C concentrations and the severity of hepatic steatosis	Atorvastatin effectively and safely reduces elevated hepatic enzyme concentrations in hypercholesterolemic patients	[78]
Fluvastatin	Rat	NASH	5 mg/kg or 10 mg/kg	Fluvastatin reduced steatosis and fibrosis scores, α-SMA protein expression, mRNA expression of pro-inflammatory and pro-fibrogenic genes in livers	Fluvastatin alleviated steatosis-induced HSCs activation and hepatic fibrogenesis through mitigating inflammation and oxidative stress	[75]
Rosuvastatin	Human	NASH	10 mg	Rosuvastatin resulted in complete resolution of NASH in 19 patients, and lipid values were normalized	Rosuvastatin could ameliorate biopsy-proven NASH and reduce the risk of vascular and liver morbidity and mortality in NASH patients	[79]
Simvastatin	Mice	NAFLD	20 mg/kg	Simvastatin restored antioxidant enzyme activity and decreased lipid peroxidation and ALE-RAGE pathway activation	Simvastatin improved microcirculatory function in NAFLD by downregulating oxidative and ALE-RAGE stress and attenuated steatosis, inflammation and fibrosis.	[86]

NAFLD, nonalcoholic fatty liver disease; NASH, nonalcoholic steatohepatitis; PPAR, peroxisome proliferator-activated receptor; TG, triglycerides; NLRP3, NOD-like receptor family pyrin domain-containing 3; LDL-C, low-density lipoprotein cholesterol; α-SMA, alpha-smooth muscle actin; HSCs, hepatic stellate cells; ALE-RAGE, advanced lipoxidation end product-receptors of advanced glycation end products.

**Table 3:** Summary of the effects of statins on the intestinal microbiota in different diseases.

Disease	Type of Statin	Study Design	Dose/Day	Intestinal Microbial Changes	Results	Reference
Hypercholesteremia	Atorvastatin	Rat	10, 15, 20 mg/kg	*Proteobacteria* increased, *Firmicutes* decreased	Intestinal microbial diversity increased	[149]
		Human	20 mg	*Faecalibacterium prausnitzii*, *Akkermansia muciniphilaa* and *Oscillospira* increased *Desufovibrio* decreased	Reduced pro-inflammatory bacteria and taxa associated with atherosclerosis formation and CVD progression	[150]
Ischemic stroke	Atorvastatin	Mice	20 mg/kg	*Firmicutes* and *Lactobacillus* increased, *Bacteroidetes* decreased	Increased fecal butyrate level, promoted intestinal barrier function	[151]
Acute coronary syndrome	Statins	Human	*/*	*Parabacteroides merdae* decreased *Bifidobacterium longum subsp. longum*, *Anaerostipes hadrus* and Ruminococcus obeum increased	Specific changes in bacterial taxa were associated with disease severity or outcomes either directly or by mediating metabolites such as fatty acids and prenol lipids	[152]
Obesity	Atorvastatin	Mice	10 mg/kg	*Bacteroides*, *Butyricimonas*, and *Mucispirillum* increased	Statins improved the inflammation associated microbiota in elderly obese mice	[153]
	Rosuvastatin		3 mg/kg 9.3 mg/kg	*Lachnospiraceae*, *Rikenella* and *Coprococcus* increased *Akkermansiaceae*, *Proteobacteria* decreased		[153,154]
NASH/NAFLD	Atorvastatin	Mice	20 mg/kg	*Mucispirillum*, *Desulfovibrio*, *Anaerotruncus* and *Desulfovibrionaceae* decreased	Increased serum TCA and depleted 3-IPA	[155]
			20 mg/kg	*Ruminococcaceae* increased	Promoted the formation of deoxycholic acid and lithocholic acid	[156]

CVD, cardiovascular disease; NASH, nonalcoholic steatohepatitis; NAFLD, nonalcoholic fatty liver disease; TCA, taurocholic acid; IPA, indolepropionic acid.
